# Movement, resting, and attack behaviors of wild pumas are revealed by tri-axial accelerometer measurements

**DOI:** 10.1186/s40462-015-0030-0

**Published:** 2015-01-22

**Authors:** Yiwei Wang, Barry Nickel, Matthew Rutishauser, Caleb M Bryce, Terrie M Williams, Gabriel Elkaim, Christopher C Wilmers

**Affiliations:** Environmental Studies Department, Center for Integrated Spatial Research, University of California, 1156 High Street, Santa Cruz, CA 95064 USA; Wildlife Computers, 8345 154th Ave. NE, Redmond, WA 98052 USA; Ecology and Evolutionary Biology Department, University of California, 1156 High Street, Santa Cruz, CA 95064 USA; Computer Engineering Department, Autonomous Systems Lab, University of California, 1156 High Street, Santa Cruz, CA 95064 USA

**Keywords:** *Puma concolor*, Accelerometer, Behavior, Random forest, Predation

## Abstract

**Background:**

Accelerometers are useful tools for biologists seeking to gain a deeper understanding of the daily behavior of cryptic species. We describe how we used GPS and tri-axial accelerometer (sampling at 64 Hz) collars to monitor behaviors of free-ranging pumas (*Puma concolor*), which are difficult or impossible to observe in the wild. We attached collars to twelve pumas in the Santa Cruz Mountains, CA from 2010-2012. By implementing Random Forest models, we classified behaviors in wild pumas based on training data from observations and measurements of captive puma behavior.

**Results:**

We applied these models to accelerometer data collected from wild pumas and identified mobile and non-mobile behaviors in captive animals with an accuracy rate greater than 96%. Accuracy remained above 95% even after downsampling our accelerometer data to 16 Hz. We were further able to predict low-acceleration movement behavior (e.g. walking) and high-acceleration movement behavior (e.g. running) with 93.8% and 92% accuracy, respectively. We had difficulty predicting non-movement behaviors such as feeding and grooming due to the small size of our training dataset. Lastly, we used model-predicted and field-verified predation events to quantify acceleration characteristics of puma attacks on large prey.

**Conclusion:**

These results demonstrate that accelerometers are useful tools for classifying the behaviors of cryptic medium and large-sized terrestrial mammals in their natural habitats and can help scientists gain deeper insight into their fine-scale behavioral patterns. We also show how accelerometer measurements can provide novel insights on the energetics and predation behavior of wild animals. Lastly we discuss the conservation implications of identifying these behavioral patterns in free-ranging species as natural and anthropogenic landscape features influence animal energy allocation and habitat use.

**Electronic supplementary material:**

The online version of this article (doi:10.1186/s40462-015-0030-0) contains supplementary material, which is available to authorized users.

## Background

One of the major logistical challenges to studying animal behavior lies in our inability to continuously observe free-ranging animals [1]. While recent technological advancements in the design and versatility of bio-logging devices (e.g., Global Positioning System (GPS) tags) have substantially improved our capacity to monitor animals, our ability to document behavior continually through time and space remains limited [2]. For example, when studying the impacts of habitat fragmentation on large carnivores, it is important to understand how landscape variables influence population connectivity and animal movement, resting, and hunting patterns [3]. Accurately discerning these behaviors at a fine scale is almost impossible from location data alone, but critical for informing conservation management decisions [4].

In the last decade, accelerometer sensors have emerged as useful tools for remotely monitoring animal behavior [5,6]. By continuously measuring body movement and posture, accelerometers allow scientists to infer the behavior and energy expenditure of the instrumented individual [7]. Accelerometers have been used to study a wide range of behavior and physiology research topics, including foraging, reproduction, activity, energy budgets, and locomotion [7,8]. While accelerometry has been used successfully to differentiate behaviors across a variety of taxa, most accelerometer-based behavioral studies have focused on marine animals including marine mammals, sea turtles, sharks, and seabirds [8].

Few studies have used accelerometers to document behavioral budgets of wild terrestrial animals. Wilson et al. [9] recently used a combination of comprehensive tri-axial accelerometer measurements and extremely fine scale GPS data to describe second-by second hunting behavior in cheetahs (*Acinonyx jubatus)*. Another study on cheetahs and one on oystercatchers (*Haematopus ostralegus*) also indicated that our understanding of wildlife behavior is enhanced by even limited accelerometer data compared to GPS data alone [5,10]. Both of these studies used short temporal segments (i.e., a few seconds) of accelerometer measurements every few minutes, calibrated with field observations to predict behavior between successive GPS locations. However, many animals cannot be observed easily in the wild and sampling behavior for a few seconds every few minutes does not necessarily reflect the animal’s primary activity during that time segment.

Here we describe the use of accelerometer measurements and observations of captive animals to predict behavior in wild pumas (*Puma concolor*). Like most large felids, pumas are cryptic animals and infrequently observed in their natural environment [11], making it difficult to document the fine-scale behavioral patterns of this species. Our primary objectives were to use continuous accelerometer measurements (sampling at 64 Hz), in combination with periodic GPS readings, to distinguish different behaviors in free-roaming pumas (*Puma concolor*), describe the accelerometer signatures of puma predation events, and determine the sufficient accelerometer sampling frequency for categorizing these behaviors.

## Results

### Behavioral measurements

We paired observations of captive pumas performing activities including resting, feeding, moving, and grooming with accelerometer measurements to build a classification algorithm to categorize those behaviors in the wild animals. Using 2 captive pumas (1 male and 1 female), we documented 2142 discrete behavioral observations, including walking, grooming, resting, feeding, and fast movement and their corresponding accelerometer measurements (Table 1). From 2010-2011, we outfitted 12 wild pumas (5 males, 7 females) with GPS collars and accelerometer sensors (Table 2). Due to mechanical or software failure in the onboard accelerometer sensors or SD cards, we removed six individuals from analyses entirely and extracted 4-26 days of accelerometer data from each of the remaining individuals (Table 2).

**Table 1:** **Total 2 second observations (N) of captive puma behaviors classified by mobility class and behavior class**

**Mobility**	**Behavior**	**N**
Yes	Low acceleration movement	564
Yes	High acceleration movement	50
No	Resting	1167
No	Eating	284
No	Grooming	77

**Table 2:** **Total accelerometer and GPS data gathered from free-ranging pumas from 2010-2011**

**Puma ID**	**Sex**	**Accelerometer days sampled**	**GPS Available**
***2***	F	26.72	Yes
***5***	M	14	Yes
***7***	F	9.42	Yes
***16***	M	16	Yes
***17***	M	14	Yes
***28***	F	4	Yes
4	M	8.23**	Yes
11	F	0.66	No
13	F	20.35*	Yes
20	F	11.2	No
27	M	0	Yes
30	F	NA**	Yes

*only two axes accurately measured. **date/time inaccurately recorded. **Bolded, italicized IDs** indicate individuals used in analyses.

### Mobility model classification of captive puma activity

**Figure 1:**
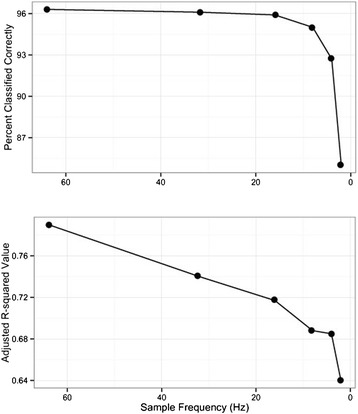
**The accuracy of predictions by the mobility model remains high until the data is sampled below 8 Hz (top graph).** The correlation between the mobility model predictions and the distance traveled declines steadily as the data is down-sampled (bottom graph).

Our mobility model, which segregated puma activity into mobile and non-mobile periods, correctly classified movements 96.17% of observed movement behaviors. The model identified Amp M (the amplitude of the dominant frequency for the magnitude of the measurements; refer to Table 3 for variable names and descriptions), DFZ (dominant frequency of the Z-axis), and SDM (standard deviation of the magnitude) as the most important variables for predicting mobility. We observed little loss of predictive power down to 16 Hz for our mobility model when we down-sampled our accelerometer measurements from 64 Hz to 32, 16, 8, 4 and 2 Hz (Figure 1).

**Table 3:** **Labels and explanations of parameters extracted from accelerometer data and used in RF models predicting puma behavioral classification** [12,13]

**Parameter**	**Label**	**Definition**
Axes	X, Y, Z	X, Y, Z axes
Magnitude	M	Square root of the sums of squares of the acceleration in the X, Y and Z axes
Dynamic body acceleration (in g)	ODBA X, ODBA Y, ODBA Z,	Mean of dynamic acceleration value along X, Y, and Z axes
Overall dynamic body acceleration	ODBA	Sum of ODBA X, ODBA Y, ODBA Z
Dominant power spectrum	Amp X, Amp Y, Amp Z, Amp M	Amplitude of dominant frequency
Dominant frequency (Hz)	DFX, DFY, DFZ, DFM	Frequency at dominant power spectrum
Standard Deviation of dynamic body acceleration and magnitude	SDX, SDY, SDZ, SDM	Standard Deviation of dynamic acceleration and magnitude in window

### Behavior model classification of captive puma activity

Our second and more comprehensive behavioral model categorized the puma’s movement behaviors into low acceleration (e.g., walk) and high acceleration (e.g., trotting and running) movements, and the non-movement behaviors into resting, eating, and grooming behaviors. This behavior model predicted captive puma resting, low, and high acceleration movement behaviors with 96.8%, 93.8%, and 92% accuracy, respectively. The model predicted feeding behavior with 65.7% accuracy but failed to detect grooming (0%) (Table 4). This behavior model identified SDM, DFZ, and SDZ as the most important variables for classification.

**Table 4:** **Cross-validation of actual (rows) and predicted (columns) behaviors of captive animals as categorized by the behavior model**

	**Feed**	**Groom**	**Rest**	**High**	**Low**	**Percent accurate**
Feed	179	0	67	0	38	63.7
Groom	20	0	54	0	1	0
Rest	26	1	1130	0	10	96.8
High	0	0	0	46	4	92
Low	24	0	9	2	529	93.8

High and low represent high and low acceleration movements.

### Model predictions of wild puma activity

We found a strong positive relationship between our model predictions of percent mobility and the distance traveled by wild pumas between successive 15-minute GPS points (*β* = 5.245 standard error = 0.174, *p* < 0.001), which further supports the model’s overall classification accuracy. Random effects were not significant.

**Figure 2:**
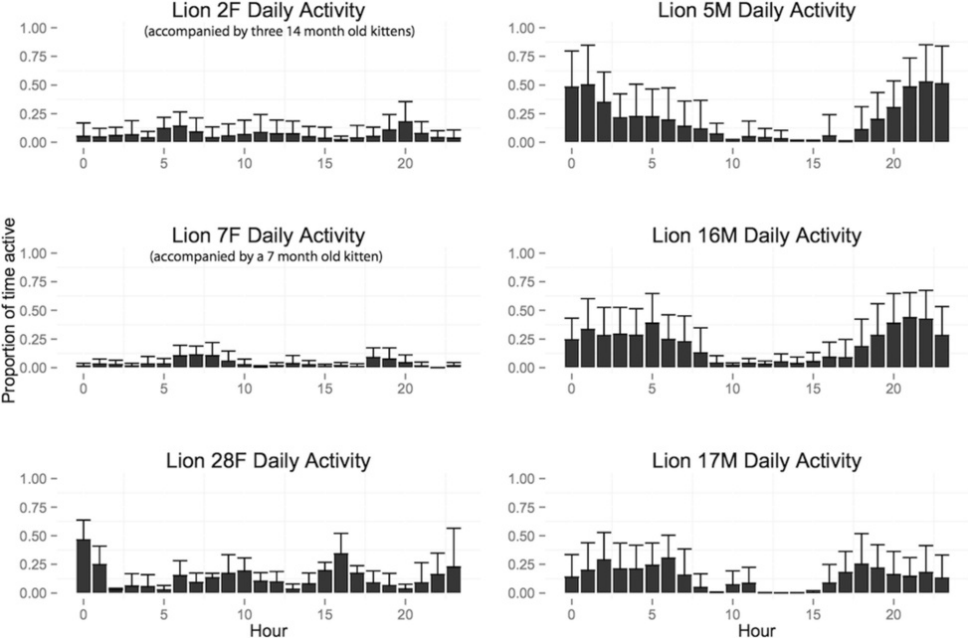
**Predicted average hourly activity across a 24-hour period for all pumas (+1 SD).** These behavioral dairies are averaged over 25.64 days (2F), 13 days (5M), 8.42 days (7F), 13 days (17M), 16 days (16M) and 4 days (28F).

**Figure 3:**
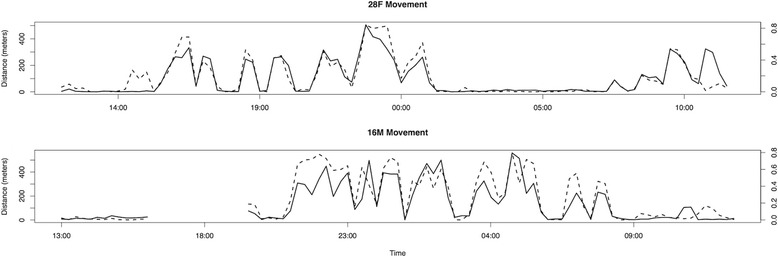
**15 minute movement distances measured directly from subsequent GPS locations (solid) and associated predicted movement activity based on accelerometry (dashed) for pumas 28F and 16M.** While we would not expect activity levels to be perfectly correlated with linear movement distances, the level or correspondence between these two measures provides a field-based assessment of the ability of accelerometer measurements to predict movement activity.

**Figure 4:**
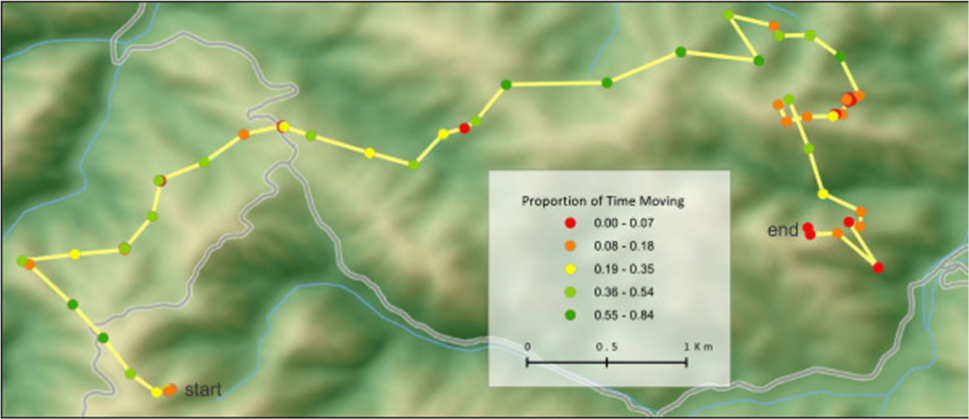
**15 minute GPS locations and associated predicted movement activity for 28F.**

Our 24-hour activity budgets for individual pumas show a general pattern of decreased movement activity in the daytime and increased activity at night (Figure 2). Males were more active than females (t = 13.37, p = 0) and more often spent over 50% of their hourly increments moving (15% for males versus 1% for females). Males were more active nocturnally (6PM to 6AM; t =15.22, p = 0) whereas females did not display a significant nocturnal movement preference (t = 0.59, p = 0.28). However, even for pumas of the same sex, individuals exhibited considerable variability in their activity. For example, 2F and 7F, both females with kittens, moved very little even during dawn and dusk, whereas 28F, a female without kittens, was much more active and even moved regularly throughout the daytime. A territorial male, 5M, exhibited one peak in activity around midnight whereas the two other males experienced a decrease in activity around midnight. When detailed GPS information was available, the actual distance traveled by pumas strongly corroborated the proportion of time we predicted movement (Figures 3 and 4).

**Figure 5:**
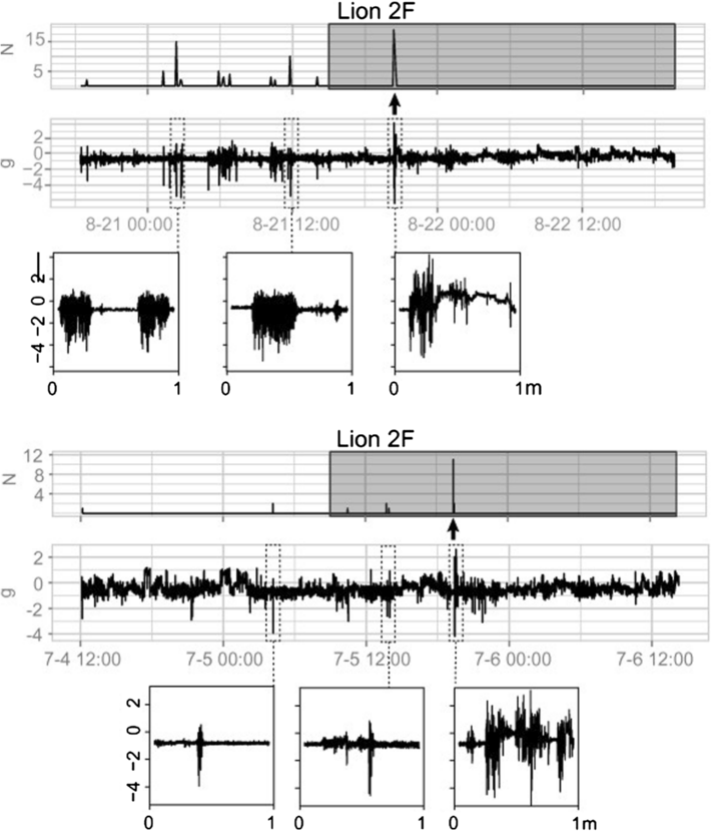
**Plots of two predation events by puma 2F.** The top panel for each plot illustrates the number (N) of high acceleration movements per minute over a period of two days. The dark grey rectangle highlights the period of time associated with the predation event as verified independently from field visits to clusters of GPS locations. The bottom panel shows the raw accelerometer measurements in units of gravity *g* for the Z-axis. The bottom inserts magnify a one-minute period of accelerometer measurements from selected large clusters to show the magnitude and duration of the acceleration during those high acceleration events. The arrow indicates when we hypothesize the kill event to have occurred.

**Figure 6:**
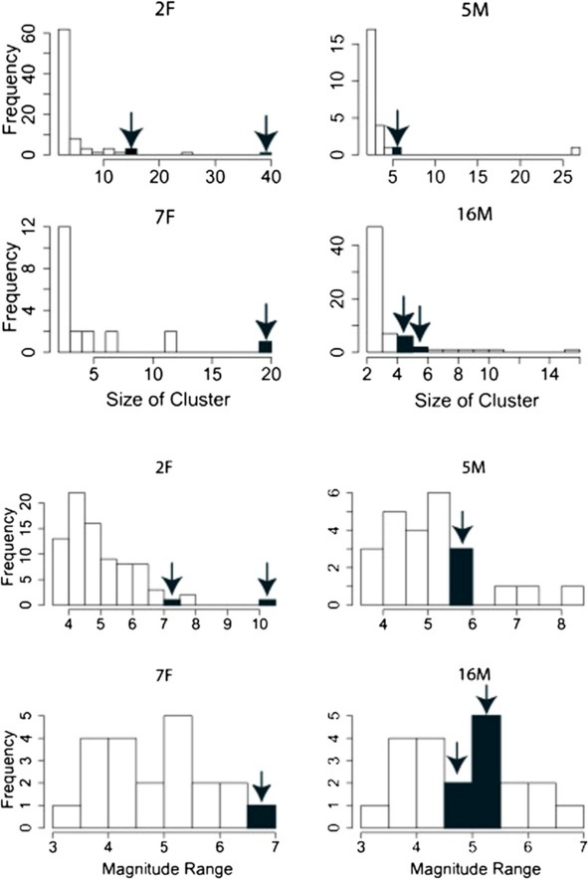
**Histograms showing how the size (top) and magnitude (bottom) of clusters of predicted high acceleration movements associated with predation events compare to those not associated with predation events for pumas 2F, 5M, 7F and 16M.** Bins containing values corresponding to verified predation events are highlighted in red and accented by an arrow. Kills by 2F and 7F at the far right of the histogram were of adult deer whereas those of 16M and 5M in the center of the histogram were of fawns.

We tested whether puma predation events, identified from GPS data, were defined by clusters of high acceleration movements. We observed a sustained cluster of high acceleration movements, as identified by the behavior model, in the time period between one GPS sampling interval before the start of a predation event through the end of the first quartile of the event (Figure 5, Additional file 1: Figure S1). When compared to other clusters of high acceleration movement, which may not necessarily be associated with predation events, 4 of our 6 potential predation events were ranked among the top 10% for cluster size (or length) and 3 of 6 for maximum magnitude (Figure 6). We recorded sustained, high acceleration movements for female puma predation incidents, likely because they were subduing large, adult deer. In comparison, the male puma predation incidents we identified consisted of fewer high acceleration movements because their targeted prey were fawns during the data collection period.

From the limited predation incidents we analyzed, we noted that the amount of time it took to kill prey was related to the age and size of both the predator and prey species. Based on the duration of the high acceleration movements, fawns were killed by males in less than one minute whereas females took over two minutes to kill large bucks and an unknown prey species.

## Discussion and conclusion

Our aim in this study was to use accelerometer measurements recorded on captive animals as a proxy to classify behavior in wild animals. Using Random Forest models, we were able to accurately predict periods of non-movement, low acceleration (i.e., stalking, walking), and high acceleration movements (i.e., trotting and running) in unobservable wild animals. This insight allowed us to better document puma movement patterns and activity levels throughout the day and to identify individual and sex differences.

**Figure 7:**
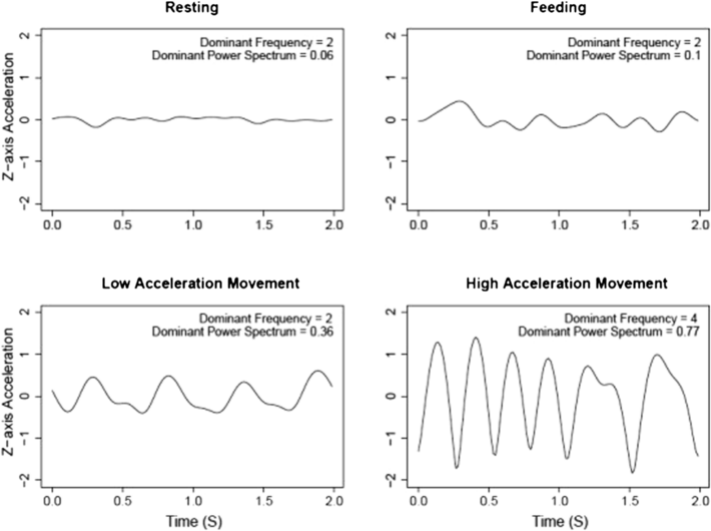
**Two-second windows of Z-axis acceleration for four behaviors with associated dominant frequencies and dominant power spectrums.**

Our model identified Amp M, DFZ, and SDM as the top ranked predictors of puma mobility. The first two variables are strongly tied to the periodicity of the movement since Amp M is the dominant power spectrum of the magnitude and DFZ is the dominant frequency of the Z-axis, which measures the heave (up-down motion) of the animal. For terrestrial quadrupeds, movement behaviors result in cyclic accelerometer patterns that are dominated by one frequency because the accelerations are primarily produced by footfalls and body movements (Figure 7) [14]. These dominant frequencies correspond to footfall patterns and can be used for biomechanical and energetics analyses [15,16]. The third parameter identified is the standard deviation of the magnitude, which is higher during mobile than non-mobile behaviors. Taken together, these parameters can clearly distinguish movement behavior, which is characterized by higher acceleration and periodicity, from non-movement activities. The tight association between footfall frequency and the dominant frequency of the accelerometer measurements bodes many promising avenues for calculating daily energetic expenditure [15].

Most current research on animal movement uses GPS or radio-telemetry collars [2], which only allow researchers to measure locations sporadically throughout each day. However, with GPS tags sampling at a low temporal resolution, it is difficult to distinguish between an animal that moves 500 meters in a straight line in a short time period from an animal that is active for longer but meanders only a short distance in a nonlinear fashion [17]. In contrast, accelerometers take near continuous measurements, thus providing fine-scale documentation of animal behavior while the instrument is activated [1]. The enhanced dataset from continuously sampling accelerometers can yield detailed information on behavior (e.g. whether the animal is traveling or potentially hunting) and energy expenditure between successive GPS fixes [15].

With continual advancements in biologging technologies, some tag designs now include GPS sensors that work synergistically with accelerometers (i.e. accelerometer-informed GPS), allowing for more flexible and intelligent GPS sampling intervals [9,17]. Such accelerometer-informed GPS tags also reduce battery consumption (thereby prolonging field deployment) by only recording GPS data points when the animal is actively moving [17]. Accelerometers could also be programmed to trigger onboard cameras like Kittycam, which was used to document domestic cat predation on wildlife [18], to more efficiently quantify hunting attempts and kills of small prey that are difficult to identify using GPS data alone. Cameras on wild animals can also be used to verify behaviors (e.g. footfall counts for energetics) predicted by models generated using accelerometer data [19].

We noted that puma predation events were associated with high acceleration movements, which were predicted by the behavior model. Although clusters of high acceleration movement occurred throughout the day, the events that we classified as kills had raw accelerometer readings with magnitudes that were generally longer and larger compared to other events (Figures 5 and 6). Additionally, there were several other prolonged high acceleration movement clusters of similar or even larger magnitudes that were not associated with identified kills. These events may have been unidentified kills or unsuccessful hunting attempts.

In our study, accelerometer sensors provided new insights on the variability and duration of predation events identified by GPS locations and confirmed by subsequent field validations. While predation by females on adult prey occurred over several minutes and included some of the highest magnitude ranges, events involving larger-bodied male pumas and smaller juvenile prey were not as extreme. More broadly, we may be able to characterize predation and attempted predation events of other medium and large bodied mammals using paired accelerometer and GPS data and corroborating these predictions with field visitations of kill site GPS clusters [3]. Combining such detailed locational and acceleration information can reveal the duration, energetic expenditure, and chase sequence of puma predation events and help scientists gain more insight about the hunting behavior of pumas [15].

Our behavior model was weak at predicting feeding events and was not able to predict grooming behaviors. This is most likely due to the complexity of identifying non-locomotive motion [7] and to the relatively few instances of these behaviors observed in the captive pumas. While grooming is a relatively unimportant behavior to identify, accurately predicting feeding events is crucial in understanding behavior and energetics. We believe these issues can be overcome in the future through a variety of innovative strategies. Studies on oystercatchers, cheetahs, and other species have used accelerometers to identify feeding bouts in terrestrial animals with some success [5,10]. However, both studies were able to observe the collared individual engaging in these behaviors and use that information to classify additional events. While we are unable to directly observe pumas in the wild, we may be able to record their feeding behavior using cameras placed at bait sites or fresh carcasses [20]. Using this information, we could then identify accelerometer sequences that correspond with feeding in the field and then use that to identify future feeding events. Additionally, we could perform more feeding trials with captive pumas using whole deer and raccoon carcasses so as to more closely mimic their dietary habits in our study area.

Integrating accelerometer technology into dataloggers has great potential for animal behavioral research and is being increasingly adopted in ecological and conservation studies [4,8]. Data derived from accelerometers can also be used to assess how animals respond behaviorally and energetically to anthropogenic influences [15,21]. For example, pumas and other large carnivores may change their overall activity levels and hunting patterns, thus impacting their caloric demands, when moving through human dominated habitats [3,22,23]. Additionally, caloric expenditure by pumas can be calculated more accurately using our accelerometer derived activity budgets and footfall frequencies than from GPS information alone [14,15,24].

Our research demonstrates that accelerometers can successfully predict movement behaviors in animals that are difficult to observe in the wild. However, more complex behaviors, such as feeding, might only be accurately identified with additional observations from captive and wild animals. Accelerometer sensors can be used with any terrestrial mammal to create a complete activity budget, catalogue behaviors, including predatory ones, and potentially measure energetic expenditure as well as foraging efficiency. We believe this ability to link behavior, spatial location, and energy expenditure has the potential to provide novel insights into how landscape structure influences the allocation of energy to different behaviors [25]. Such information would be valuable for conservation and management issues by revealing the detailed responses of individual animals to their surrounding landscape.

## Methods

### Study species and area

Pumas are territorial, apex predators that live in diverse habitats throughout the Americas [26]. Individuals are primarily nocturnal and solitary, although females will typically raise and accompany cubs for 15-21 months after birth. In our study area in the Santa Cruz Mountains of California (37° 10.00’ N, 122° 3.00’ W), pumas primarily feed on black-tailed deer (*Odocoileus hemionus columbianus*) but occasionally on other species, including wild boars (*Sus scrofa*), raccoons (*Procyon lotor*) and domestic cats [3].

Our 1,700 km^2^ study area encompasses a diverse landscape ranging from dense, urban development to large tracts of intact and relatively undisturbed native vegetation primarily comprised of redwood and Douglas fir, oak woodland, or coastal scrub communities. It is bisected by a large freeway and further crisscrossed by numerous smaller roads providing access to rural houses and developments. The climate is Mediterranean, with precipitation concentrated between November and April. Elevation ranges from sea level to 1155m.

### Captive animal data collection and analysis

We used custom-built collars [15,27] equipped with a tri-axial accelerometer sampling continuously at 64Hz to monitor behavior in captive and wild pumas. The tri-axial accelerometer was mounted such that the x-, y-, and z- axes were parallel to the anterior-posterior, the transverse, and the dorsal-ventral planes of the animal, respectively. Captive pumas were housed and trained by the Colorado Parks and Wildlife (Foothills Wildlife Research Facility) [15]. We outfitted one adult male and one adult female puma with a test accelerometer collar during training sessions. We observed the collared animals and recorded their behaviors both manually and with a video camera. We conducted 1-2 trials per animal during two different visits, totaling 2 hours and 40 minutes of recorded behavioral observations for collar-outfitted captive pumas. At the end of each visit, we retrieved the collar and downloaded the data.

We reviewed all recordings of captive pumas and categorized behaviors into mobile (e.g., slow movement, fast movement) or non-mobile (e.g., resting, feeding, grooming) activities. We divided observations into 2-second segments encompassing only one behavior type and extracted the corresponding accelerometer data (Figure 7). Animal activities were constrained into the above 5 classifications, and we did not catalogue transitions between behaviors since they occurred very quickly (≤1s). We chose 2-second windows because this duration accommodated at least 2 full strides of the animal within the 20 m test track used for captive puma filming and calibration trials.

To compare across collars we converted all accelerometer data into units of *g* (1 *g* = 9.8 m s^−2^) using tag specific calibration values derived prior to deployment. The process of calibrating the accelerometers consists of gently tumbling the collar and measuring the body-frame output of the accelerometer triad [28]. In the case of a perfectly calibrated accelerometer, the locus of points would all be attached to a sphere centered at the origin with a radius of 1*g*. Due to null shift errors, the sphere is centered off the origin, and scale factor errors transform the sphere into an ellipsoid. We developed a custom MATLAB 8.0 (The MathWorks, Inc., Natick, Massachusetts, United States) script to extract the null shift and scale factor errors by fitting an ellipse to the data two axes at a time, and combining the resultant parameters.

Accelerometer measurements were deconstructed into static and dynamic components [7]. Static acceleration relates to the inclination of the accelerometer with respect to the earth’s gravitational field and thereby reflects the posture of the animal. Dynamic acceleration relates to changes in velocity resulting from patterns of locomotion and generally reflects the movement of the animal. We subtracted static acceleration from collar measurements using 2-second windows [29], and then extracted 16 predictor variables from the three-accelerometer axes and the magnitude from each 2-second segment of dynamic acceleration (see Table 1). We also applied a Fast Fourier Transform to accelerometer measurements to extract the dominant frequency and dominant power spectrum values of each behavior.

We selected Random Forests (RF) [30] as our modeling tool to predict unobserved behaviors in wild animals based on measurements of observed behaviors in captive animals. RF is a relatively novel and powerful machine learning tool that works well for non-linear and complex ecological data not easily fitted by traditional methods such as generalized linear models [31]. RF also makes it possible to make accurate predictions from datasets with correlated variables and to compare conditional variable importance measures, which identify the extent to which specific predictor variables influence classification accuracy [31]. A higher measure of variable importance indicates that the variable exerts greater influence on the response relative to other predictors with lower values [32].

Our first model (mobility model) segregated mobile from non-mobile behavior. To build our model using RF, we fit 500 classification trees to a randomly selected subsample (n = 1000, without replacement) of captive puma data using a random subset of 5 predictor variables for each split in the tree [32,33]. Predictions made by all trees for each observation were then tallied, with classification assigned by the majority result and ties decided randomly. Model prediction accuracies were calculated by comparing predicted and actual classifications. We then obtained unbiased variable importance estimates using a permutation procedure described in Strobl [32]. We built our second model (a more comprehensive behavior model) using the same methodology to predict five classes of behaviors: low acceleration movement (e.g., walking), high acceleration movements (e.g. trotting, running), resting, eating, and grooming. We fit all of our models and calculated variable importance estimates using the Party package [34] in the R statistical program (vers. 2.15.1) [35].

We created down-sampled datasets of accelerometer data at 32, 16, 8, 4, and 2 Hz to explore the influence of sampling frequency on model prediction accuracy. Using the down-sampled datasets, we built additional mobility models and assessed model accuracy as described previously.

#### Wild animal data collection and analysis

We captured wild pumas (5 males, 7 females) from 2010-2012 using trailing hounds, cage traps, or leg hold snares as described in Wilmers et al. [3]. Each animal was tranquilized using Telazol and outfitted with an off-the-shelf GPS/VHF collar (Vectronics Aerospace GPS PLUS model) combined with the custom-built archival 3-axis accelerometer tag [27], which was incorporated into the battery casing (total collar weight = 480 g) Data collected by the accelerometer were recorded in an onboard 8GB microSD card, which is capable of storing more than 200 days of accelerometer measurements.

We programmed each collar to acquire a GPS fix every 4 hours and had a mean fix rate of 86% (±1%) from 4 or more satellites. For animals captured prior to April 2011, we programmed accelerometers to record at a duty-cycle of 2 weeks on, 4 weeks off commencing immediately upon capture. While accelerometers were recording, the collars were programed to acquire additional GPS fixes at 5-minute intervals between 8PM and 9PM local time for one week (GPS intensive sampling period). After April 2011, we programmed accelerometers to operate at cycles of two consecutive days every week beginning 5 days after the animal was captured to extend battery life while optimizing data collection. When accelerometers were recording, collars recorded additional GPS locations every 15 minutes during a 24-hour period from noon to noon (GPS intensive sampling period). We retrieved all collars either during a recapture of the animal (n = 8) or following its death (n = 4; 2 depredations, 2 unknown causes). The Animal Care and Use Committee at UC Santa Cruz approved all animal-handling procedures (IACUC Protocol #Wilmc1101).

We downloaded all available accelerometer data and removed the first 24 hours of data following anesthesia. We then converted accelerometer data into units of *g* as described in the previous section.

We used our captive puma-derived mobility and behavior models to predict free-ranging puma behavior from accelerometer data obtained from wild puma collars that collected at least one day of both accelerometer and GPS data. Because we could not observe behavior in wild animals, we tested the accuracy of our mobility model’s predictions by fitting a linear mixed effects model to GPS data using the lme4 package [36]. Specifically, we tested whether our mobility model predictions were positively correlated with the distance traveled by pumas between GPS points. We used the distance between successive 15-minute GPS points as our response variable and treated our model-predicted percentage of time spent moving as a fixed effect with puma ID as a random effect. We expected that longer-distance GPS movements would be correlated with a higher percentage of model-predicted movement activity, and that this relationship might vary by individual pumas. For example, during a 15-minute gap between successive GPS points, we used the mobility model to predict the percentage of time the puma was actively moving. If we mostly predicted movement, we would expect that the distance between the two GPS points to be generally larger than if we mostly predicted non-movement.

We constructed 24-hour movement budgets for all pumas to document the proportion of time pumas spent moving throughout the day. To determine the proportion of time spent moving for each one-hour period (e.g., 1AM to 2AM), we calculated the number of increments during which we predicted mobile activity and divided that by the total number of predictions we recorded between those time periods. From our behavior model, we generated 24-hour behavioral budgets for our five behavior classes.

Using the results from our behavior model, we tested whether predation events were associated with periods of high acceleration movement. We used six feeding events by four pumas on five deer and one unknown species to examine the corresponding high accelerometer movements. When feeding, pumas generally remain with the carcass over several GPS acquisitions. We used this information to estimate the duration of the feeding event as the interval of time between the first and last GPS location at the kill site. We also added the four-hour interval prior to the first GPS location associated with the site to the feeding duration since it is possible that the puma made a kill during this period of time.

Five of these feeding events were visited and verified by field personnel, and one was classified as a kill with 80% probability using a predation model developed by Wilmers et al. [3]. From observations of captive puma behavior, we know that faster and more intense movements, such as running and jumping, lead to accelerometer readings with ranges spanning 3-5 g or more. We expected that predation events would be associated with clusters of high acceleration movements as pumas attack and wrestle with their prey. To screen for possible predation events, we identified clusters of high acceleration movements with a magnitude range exceeding 3.4 *g,* or two standard deviations above the average and calculated the duration and the maximum magnitude of the event. We defined a cluster to be two or more successive high acceleration movements separated by no more than three minutes between consecutive behaviors. We expected that if high acceleration movements were associated with predation events, we would see a cluster of high acceleration movements in the first quartile of each predation event. Compared to non-predation clusters of high acceleration movements, we also expected the size and maximum magnitude of the cluster representing a potential predation event to be in the top 10% of those measurements across all clusters.

## Additional file

Additional file 1: Figure S1. Plots of predation events by pumas 5 M, 7 F, and 16 M, analogous to Figure 2. The top panel for each plot illustrates the number (N) of high acceleration movements per minute over a period of two days. The dark grey rectangle highlights the period of time associated with the predation event as verified independently from field visits to clusters of GPS locations [3]. The bottom panel shows the raw accelerometer measurements in units of gravity *g* for the Z-axis. The bottom insets magnify a one-minute period of accelerometer measurements from selected large clusters to show the magnitude and duration of the acceleration during those high acceleration events. The arrow indicates when we hypothesize the kill event to have occurred [15].
