# Supplementary figures and images for: Movement, resting, and attack behaviors of wild pumas are revealed by tri-axial accelerometer measurements
Source: Mov Ecol. 2015 Jan 22;3(1):2. doi: 10.1186/s40462-015-0030-0 (PMC4337468; doi:10.1186/s40462-015-0030-0)

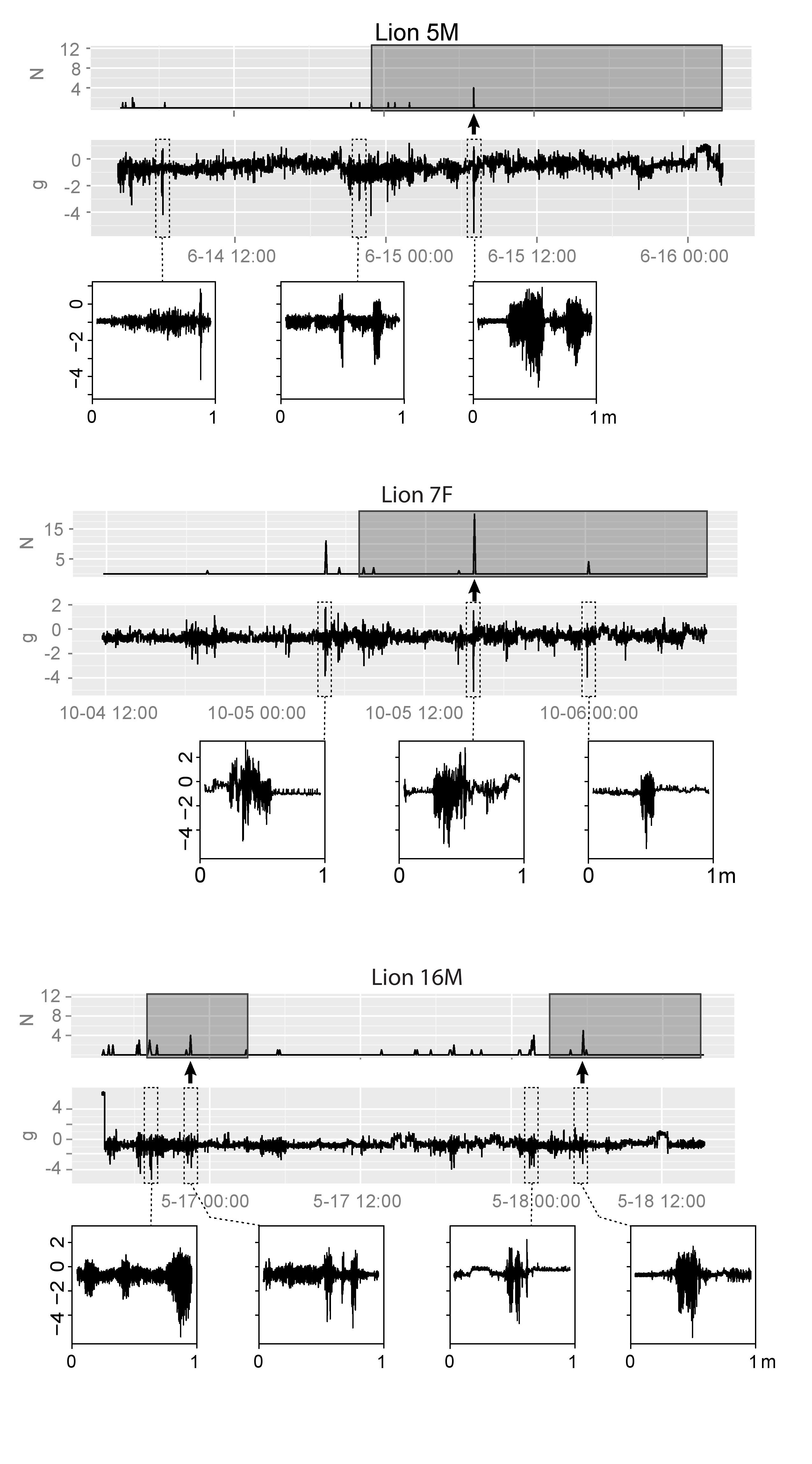

Supplement: Additional file 1: Figure S1. — Plots of predation events by pumas 5 M, 7 F, and 16 M, analogous to Figure 2. The top panel for each plot illustrates the number (N) of high acceleration movements per minute over a period of two days. The dark grey rectangle highlights the period of time associated with the predation event as verified independently from field visits to clusters of GPS locations [3]. The bottom panel shows the raw accelerometer measurements in units of gravity g for the Z-axis. The bottom insets magnify a one-minute period of accelerometer measurements from selected large clusters to show the magnitude and duration of the acceleration during those high acceleration events. The arrow indicates when we hypothesize the kill event to have occurred [15]. [file 40462_2015_30_MOESM1_ESM.jpeg]
